# Good Advice to Girls

**Published:** 1891-11

**Authors:** 


### GOOD ADVICE TO GIRLS.

Scarcely a day passes without its newspaper story of some young woman who met a man so interesting that she thought she couldn't live without him, so she married him in haste and afterward learned that he was an ex-convict or a brute, or already had a wife or two from whom he had separated without the formality of a legal divorce. In such cases the blame is laid upon the man, who generally deserves more abuse than he gets. But, girls, look at the matter seriously a few minutes and see if the trouble might not have been avoided if you had not been in too much of a hurry.

Marriage means partnership for life ; decrees of divorce are merely exceptions that prove the rule. Would any man enter into a business partnership with as little knowledge of the other party as you seem satisfied with ? Well, no—not unless he were a sweet souled lunatic. Talk is cheap, girls ; it can be made to order as fast as the tongue can run, especially when there is a pretty face to inspire it and two ears willing to receive it.

Don't fear that some other girl will get the fellow unless you secure him at once. A fish that any one can catch isn't worth throwing a line for. Play him to find out whether he amounts to any thing. If he becomes impatient and dashes away, why, follow Dogberry—thank God that you're rid of a knave.—*Truth.*

